# Epidural lipomatosis and congenital small spinal canal in spinal anaesthesia: a case report and review of the literature

**DOI:** 10.1186/1752-1947-3-128

**Published:** 2009-11-16

**Authors:** Per Flisberg, Owain Thomas, Bo Geijer, Ulf Schött

**Affiliations:** 1 Department of Intensive and Perioperative Care, Lund University Hospital, 22185 Lund, Sweden; 2 Department of Radiology, Halmstad Central Hospital, Halmstad, Sweden

## Abstract

**Introduction:**

Complications after lumbar anaesthesia and epidural blood patch have been described in patients with congenital small spinal canal and increased epidural fat or epidural lipomatosis. These conditions, whether occurring separately or in combination, require magnetic resonance imaging for diagnosis and grading, but their clinical significance is still unclear.

**Case presentation:**

A 35-year-old Caucasian woman who was undergoing a Caesarean section developed a longstanding L4-L5 unilateral neuropathy after the administration of spinal anaesthesia. There were several attempts to correctly position the needle, one of which resulted in paraesthesia. A magnetic resonance image revealed that the patient's bony spinal canal was congenitally small and had excess epidural fat. The cross-sectional area of the dural sac was then reduced, which left practically no free cerebrospinal fluid space.

**Conclusion:**

The combination of epidural lipomatosis of varying degrees and congenital small spinal canal has not been previously discussed with spinal anaesthesia. Due to the low cerebrospinal fluid content of the small dural sac, the cauda equina becomes a firm system with a very limited possibility for the nerve roots to move away from the puncture needle when it is inserted into the dural sac. This constitutes risks of technical difficulties and neuropathies with spinal anaesthesia.

## Introduction

Epidural lipomatosis is the presence of excessive fatty tissues within the epidural space of the spinal canal. First described as causing spinal cord compression, epidural lipomatosis has long been associated not only with Cushing's syndrome, exogenous intake of corticosteroids and obesity, but also more recently with protease inhibitor treatment in patients with HIV [1-4]. Patients with epidural lipomatosis may be asymptomatic. However, the disease can also manifest as mild back pain, radicular symptoms or neurogenic claudication with decreased strength, sensation and reflexes depending on its severity and the vertebral level involved. Alterations in bowel and bladder functions are unusual.

A system for grading the severity of lumbar epidural lipomatosis (LEL) was introduced by Borré *et al*. in 2004 [1] and reevaluated by Pinkhardt *et al*. in 2007 [2]. The LEL grade is determined by the proportions of epidural fat occupying the spinal canal and the dural sac. Borré's grades range from LEL 0 (no lipomatosis) to LEL III (severe lipomatosis). Borré grade 0 or normal was defined as epidural fat occupying less than 40% of the canal width and 150% of the dural sac width; grade I was 50% of both the canal and the dural sac; grade II was 50% to 75% of the canal width and 100% to 150% of the dural sac; and grade III was 75% of the canal width and 30% of the dural sac.

Known spinal compression or stenosis is a strong contraindication to neuraxial blockade as postoperative magnetic resonance image (MRI) scanning has shown spinal stenosis in patients with neuropathies after regional anaesthesia [5]. There are several causes of lumbar spinal stenosis. Degenerative changes usually manifest after the sixth decade of life while excessive scoliosis or lordosis may narrow the spinal canal from earlier ages. Congenital lumbar spinal canal stenosis is a developmental defect [6].

Several factors contribute to the risk of traumatic needle damage to the conus medullaris during subarachnoid or combined subarachnoid and epidural anaesthesia [7]. Identifying the correct lumbar interspace is in itself difficult [8], tethered cords may reside at a level lower than what is usually expected, and congenital variations where the conus may stretch down to L4 and L5 can occur [9]. Cutting needles (Touhy, Quinke) may increase the risk of nerve damage but even pencil-point needles may cause harm, requiring both a greater force for dural sac penetration and a deeper insertion of the tip to introduce the orifice into the subarachnoid space. Paraesthesia and pain upon injection of local anaesthetics are likewise associated with nerve trauma.

We describe in this report a patient who experienced persisting unilateral sensory and motor neurological deficits after subarachnoid anaesthesia and who was also later diagnosed with epidural lipomatosis and congenital small spinal canal.

## Case presentation

A semi-urgent Caesarean section was carried out on an obese (body mass index 45, weight 130 kg, height 170 cm) 35-year-old Caucasian woman due to cephalopelvic disproportion. She had no previous neurological problems. Subarachnoid anaesthesia was performed by a medial approach at L3 and L4 vertebrae with the patient in the sitting position. A long 25 gauge Quincke (Whitacre^®^) needle was used to inject 1.8 ml hyperbaric bupivacaine (5 mg/ml) and satisfactory sensory anaesthesia was achieved up to a level equivalent to T5. The attending anaesthetist did not document any difficulties, but the patient later reported that several attempts were needed to obtain correct positioning of the needle and that she had experienced one minor episode of paraesthesia in her right leg. The Caesarean section was uneventful but the patient complained postoperatively that the spinal anaesthesia had not yet worn off. However, no immediate action was considered necessary.

Two days later, the patient could still not support her right leg. A neurological examination verified unilateral neuropathy affecting the patient's right lumbar roots of L4 to S1: knee extension (L3 to L4), ankle dorsiflexion (L4) and foot eversion (L5 to S1) were severely weak; hip flexion (L1 to L2) was slightly weak, and there was sensory loss for pinprick and cold sensation on the lateral aspect of the right lower leg (L5), the lateral side of the foot (L5 to S1) and the perineum (S2 to S3). The following were unaffected: plantar flexion (S1 to S2), foot inversion (L4 to L5), knee flexion (S1), gluteal function (L4 to L5, S1 to S2) and sphincter tone (S2 to S4). All the patient's deep tendon reflexes were normal.

**Figure 1:**
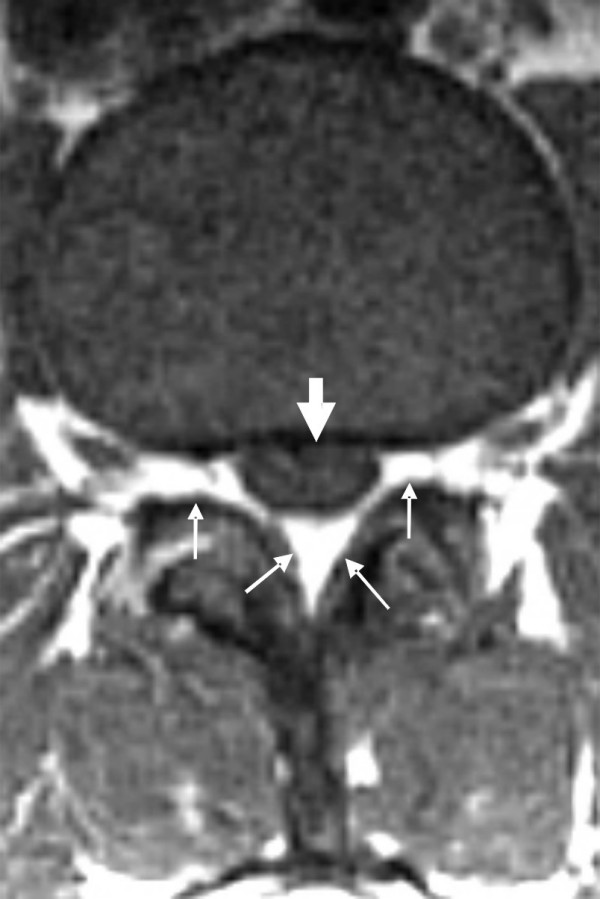
**A T1-weighted cross-sectional magnetic resonance image at the level of lumbar puncture (T2-weighted cross-sectional images were not obtained)**. The bony spinal canal is congenitally relatively small. The cross-sectional area of our patient's dural sac was 80 mm^2^. With excess epidural fat of LEL 2 according to Borré *(small arrows)*, the dural sac completely outlines the bundle of nerve roots (greyish appearing, *large arrow*) leaving no free cerebrospinal fluid space, which would have appeared nearly black. These nerve roots are not completely free to move away when a needle is inserted into the dural sac.

An MRI scan revealed a congenital small bony spinal canal combined with a degree of lipomatosis equivalent to LEL 2 according to Borré's grade. The sagittal diameters of the patient's spinal canal and dural sac were 16 mm and 10 mm, respectively, while her epidural fat and/or spinal canal index was 10/16 = 62.5%. Her dural sac was considered small (6 mm), leaving practically no free space for the cerebrospinal fluid (CSF) (Figure 1). The cords of the patient's cauda equina were compressed into a tight bundle. After neurosurgical liaison, the examining neurologist recommended watchful waiting including electromyography (EMG) and electroneurography (ENG) after six weeks. These investigations did not detect any lumbosacral nerve root pathology and the patient has slowly improved. Eight months later, however, she was still experiencing lower back pain and weakness in her right leg.

## Discussion

Spinal cord and nerve root injury after neuraxial blocks may be caused by compression, ischemia, needle and/or catheter trauma, toxic reactions to local anaesthetics, or a concurrent neurological disease. Compression may be caused by a variety of factors such as bone disease (spinal stenosis, spondylolisthesis), disc herniation, hypertrophy of ligamentum flavum, extramedullary hematopoiesis, blood, abscesses, cysts, tumors and epidural lipomatosis.

Epidural lipomatosis is believed to be an uncommon disorder since only approximately 100 cases have been discussed in the literature. The prevalence of epidural lipomatosis is unknown and the diagnosis of epidural lipomatosis relies heavily on computed tomography or MRI [1,2]. Borré [1] found that all patients with a lipomatosis degree of LEL 3 were symptomatic of peripheral nerve symptoms. However, many patients with epidural lipomatosis of the levels LEL 1 and LEL 2 may be asymptomatic. The clinical significance with respect to primary affection of the nerve roots is not clear [2], but logically, and from our case, we can see that the condition constitutes an increased risk of complications during the administration of lumbar anaesthesia.

A recent study reveals that weight rather than body habitus was associated with the deposition of epidural fat, and that overall obesity was unrelated to the amount of epidural fat deposited [10]. The distribution of epidural fat in epidural lipomatosis may vary depending on the region involved in the cases studied: thoracic region 46%, lumbosacral region 44%, and both regions 10%.

**Figure 2:**
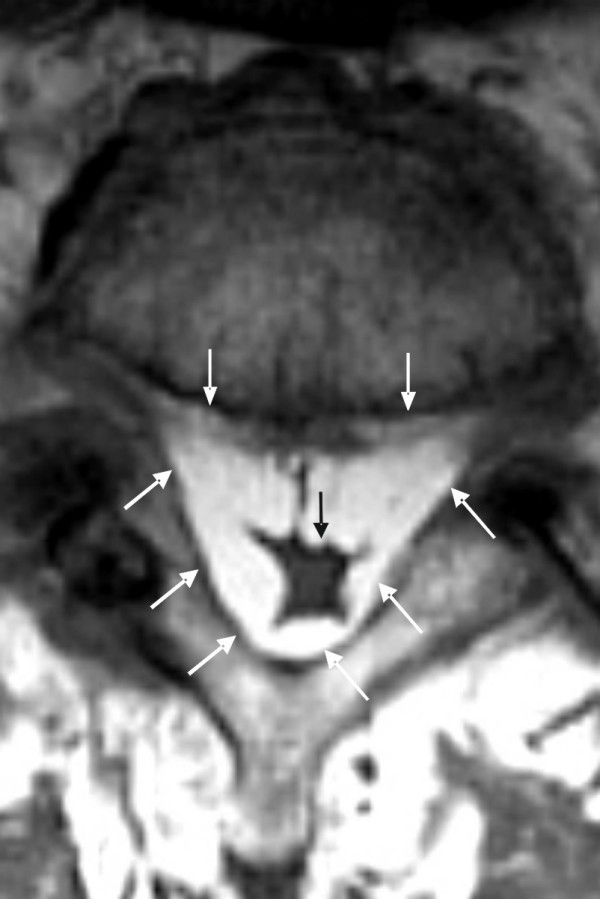
**Magnetic resonance images of a true epidural lipomatosis**. A T1-weighted cross-sectional image of a true epidural lipomatosis at the lumbar level of LEL 3 (according to Borré). There is a large intraspinal space, which is filled by an excessive amount of epidural fat *(white arrows) *and squeezes the cerebrospinal fluid away so that the dural sac *(black arrow) *completely surrounds the bundle of nerve roots and leaving no free intradural cerebrospinal fluid volume (dural sac cross-sectional area of only 48 mm^2^). These nerve roots are not completely free to move away when a needle is inserted into the dural sac.

The presence of excess epidural fat may lead to certain problems. In one case, the insertion of an intrathecal baclofen pump failed and the magnetic resonance image exposed a thoracolumbar lipomatosis profoundly compressing the dural sac, thus creating significant spinal cord atrophy [11]. A laminectomy and a removal of epidural fat had to be undertaken in order to facilitate the placement of the intrathecal catheter. Such a dural sac compression is illustrated in a case of another patient of ours (Figure 2), with true epidural lipomatosis of Borré grade III (sagittal diameters of spinal canal 24 mm, epidural fat 18 mm, and dural sac 6 mm; with an epidural fat and/or spinal canal index of 18/24 = 0.75%) seen in a T2-weighted image and a dural sac cross-sectional area of only 48 mm^2^. In another patient with the combination of congenital lumbar spinal stenosis and epidural lipomatosis with a dural sac cross-sectional area of 77 mm^2^, an epidural blood patch caused an acute spinal pain that was probably secondary to increased dural sac compression and increased pressures within the sac [12].

The risk for extensive blocks with spinal anaesthesia in patients with increased abdominal pressure such as obesity or pregnancy has been discussed by Hogan *et al*. [13]. The mechanism they suggested was a reduction of the CSF volume due to the inward movement of soft tissues through the vertebral foramen that negated the risk of epidural fat.

The definition [14] of absolute spinal stenosis at L3 and L4 levels is usually a dural sac area size of 70 mm^2 ^to 80 mm^2^. On the other hand, relative stenosis is usually at 90 mm^2 ^to 100 mm^2^. Magnetic resonance images may reveal significantly decreased pedicle length (< 6.5 mm) with a decreased cross-sectional spinal canal area (< 213 mm^2^) in patients with symptoms. The normal ovoid shape of the spinal canal is changed to a flattened appearance with a decreased anterior-posterior diameter of < 10 mm with a reduced dural sac cross-sectional area of < 77 ± 13 mm^2^. In our patient, the dural sac cross-sectional area was 80 mm^2 ^(Figure 1). She had no previous neurological symptoms. Therefore, asymptomatic younger patients with congenitally small spinal canals may be at risk for developing symptoms after minor degenerative processes that cause a further narrowing of the spinal canal.

**Figure 3:**
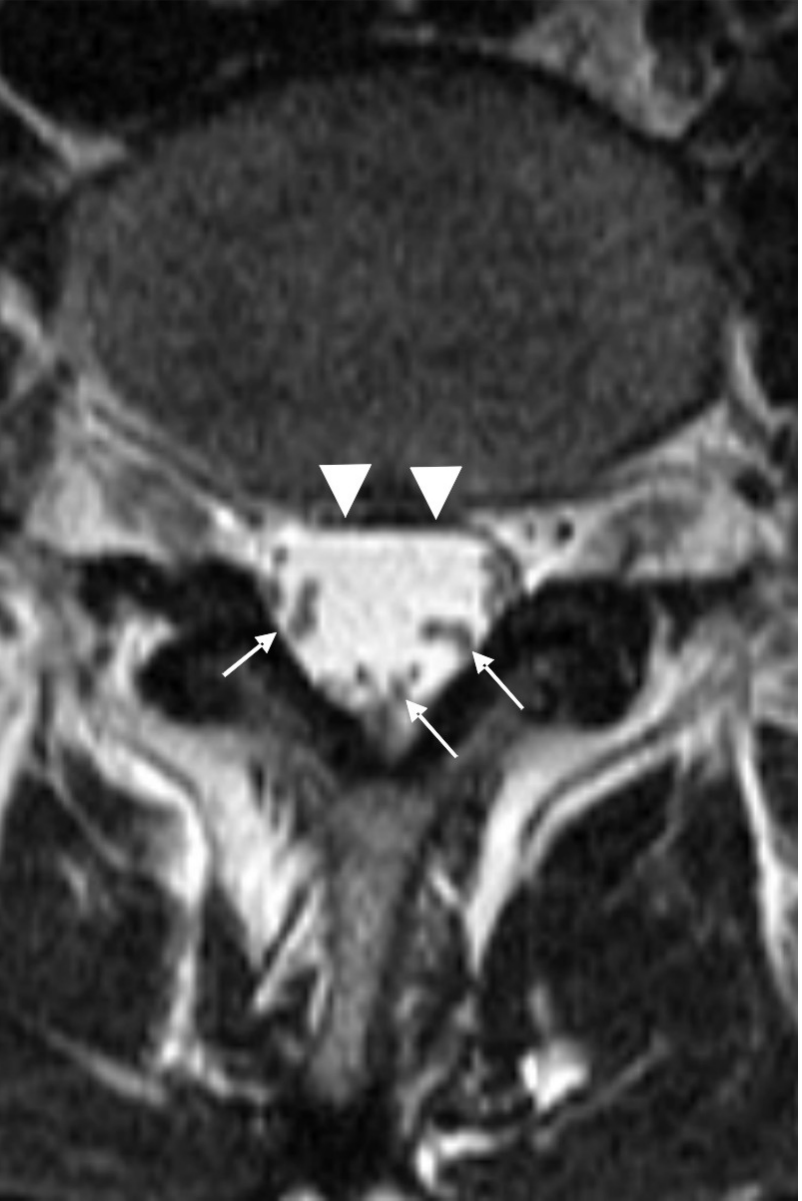
**Magnetic resonance images of a normal lumbar spine**. Cross-sectional magnetic resonance images of a normal lumbar spine with dural sac cross-sectional area of 180 mm^2 ^and LEL 0 (according to Borré). On the T2-weighted image, the cerebrospinal fluid appears nearly white and the nerve roots are more easily seen in the large cerebrospinal volume than on a T1-weighted image. The nerve roots are free to move away in the cerebrospinal fluid space when a needle is inserted into the dural sac.

The cauda equina is normally mobile within the CSF [15] and can readily move about as the patient changes her posture. This is illustrated in the case of another patient of ours (Figure 3), with a dural sac cross-sectional area of 180 mm^2 ^(sagittal diameters of spinal canal at 16 mm, epidural fat at 3 mm and dural sac at 13 mm, with an epidural fat and/or spinal canal index of 3/16 = 19% (LEL 0 according to Borré). This large dural sac and excess volume of CSF usually guarantees the correct placement of the spinal needle and thereby minimizes the risk of accidental nerve injury via direct needle trauma. An abnormal anatomy caused by epidural lipomatosis and/or small spinal canal, a reduced CSF volume, which can also occur in pregnancy due to increased intra-abdominal pressure, and a less movable cauda equina might all accidentally lead to nerve injury after the administration of spinal anaesthesia, as happened in our patient (Figure 1). Our patient had unilateral lumbosacral nerve affection that corresponded to the lumbar puncture site and the resulting paraesthesia. Post-spinal and obstetric neuropathies are usually transient but paraesthesia and pain at injection may increase the risk for long-term damage. Electromyography and electroneurography yielded normal results six weeks after the patient's Caesarean section, although she still felt a weakness in her leg six months after the operation. Electromyography only measures large nerve fibre signals and it may take up to three weeks before a nerve lesion due to nerve injury can be confirmed [10].

## Conclusion

This patient's case illustrates that a small dural sac secondary to an increased content of epidural fat and/or a congenital small spinal bony canal may be a potentially dangerous combination in conjunction with spinal anaesthesia. Difficulties in identifying the dural sac due to the absence of spinal fluid return may lead to multiple spinal punctures, thereby increasing the risk of nerve damage and the development of a less movable and compressed cauda equina. We believe that these aspects of spinal anaesthesia have not been previously discussed in the literature.

## Abbreviations

CSF: cerebrospinal fluid; EMG: electromyography; ENG: electroneurography; LEL: lumbar epidural lipomatosis; MRI: magnetic resonance imaging.

## Consent

Written informed consent was obtained from the patient for publication of this case report and any accompanying images. A copy of the written consent is available for review by the Editor-in-Chief of this journal.

## Competing interests

The authors declare that they have no competing interests.

## Authors' contributions

US was the physician who principally attended the patient. BG, the radiologist on call, arranged and analyzed the figures and legends cited in the manuscript. PF, US and OT drafted the manuscript. All authors read and approved the final manuscript
